# 222 Does Chlorhexidine Bathing Contribute to Chlorhexidine Resistance?

**DOI:** 10.1017/ash.2026.10602

**Published:** 2026-06-23

**Authors:** Ivis Call Rosado, Rachel Guran

**Affiliations:** 1 Memorial Healthcare System

## Abstract

**Background:** High-level disinfection (HLD) of ultrasound probes is a required patient safety and regulatory practice. Automated vaporized hydrogen peroxide (VHP) systems, widely implemented for HLD since 2011, involve sizable capital costs, substantial maintenance requirements, and lengthy probe turnaround times. Chlorine dioxide foam has been used for ultrasound probe HLD in Europe since the late 1990s and received U.S. Food and Drug Administration clearance in 2023, consistent with the ANSI/AAMI ST58 standard. Evidence describing operational impact and feasibility in U.S. outpatient practice remains limited. Objective: To compare workflow and probe turnaround times between two ultrasound probe HLD methods and to evaluate the feasibility of chlorine dioxide foam implementation, including staff training requirements and adherence to reprocessing protocols. **Methods:** From October 2025 to January 2026, we conducted a quality improvement study examining the transition from automated VHP to manual chlorine dioxide foam for transducer HLD in an outpatient urology practice. A total of six registered nurses and four medical assistants participated in the study. Training provided to staff included manufacturer-provided online training, 1:1 in-person instruction, and direct observation to confirm competence with the new reprocessing protocol. Workflow steps for both HLD methods were mapped and compared. Outcomes measured included probe turnaround time (defined as the interval from immediate post-use handling to readiness for subsequent clinical use); number of workflow steps; feasibility of protocol-specific staff training; and adherence to the reprocessing protocol. **Results:** Each of the methods had a comparable number of workflow steps. Average probe turnaround time for chlorine dioxide was four minutes, while the automated VHP system required 14–15 minutes. Most participants completed manufacturer-provided online training. All participants received 15 minutes of 1:1 in-person instruction and direct observation. Overall, direct observation demonstrated high adherence to the reprocessing protocol. The manual foam process was integrated into routine clinical workflows without disruption to patient care. **Conclusion:** In a single outpatient urology practice, manual chlorine dioxide foam disinfection was feasible to implement and integrated into existing workflows with considerably shorter probe turnaround time while maintaining staff adherence. Results informed our policy on workflow steps, training, and documentation. Observations were conducted over a limited period, and findings may not be generalizable to other clinical settings.

